# The impact of the COVID-19 pandemic on telework and short sickness absences among Finnish knowledge workers

**DOI:** 10.3389/fpubh.2025.1683731

**Published:** 2025-11-19

**Authors:** Annina Ropponen, Annu Haapakangas

**Affiliations:** 1 Finnish Institute of Occupational Health, Helsinki, Finland; 2 Division of Insurance Medicine, Department of Clinical Neuroscience, Karolinska Institute, Stockholm, Sweden

**Keywords:** telework, sick leave, longitudinal, occupational health, well-being

## Abstract

**Introduction:**

Longitudinal studies are rare for associations between telework and sickness absence (SA). This prospective cohort study aimed to investigate short (1–3 days) SA from 2019 to 2023 among a cohort of Finnish knowledge workers and the role of telework in short SA.

**Methods:**

Complete employer-owned register data on SA and telework were available from four organizations for 2019–2023, comprising 924 employees. Weekly means of telework days/week and short (1–3 days) SA days were calculated and analyzed using conditional Poisson with fixed-effects to obtain incidence rate ratios (IRRs) with 95% confidence intervals (CIs).

**Results:**

The levels of short SA were low from 2019 to 2023, while telework increased at the start of the COVID-19 pandemic and remained at an elevated level. In a fully adjusted model, each 1-day increase in telework was associated with a higher likelihood of short SA (IRR 1.35, 95%CI 1.22, 1.50).

**Conclusion:**

Telework has become a central way of working among Finnish knowledge workers since the COVID-19 pandemic, while shortSA has remained at a low level. A higher number of weekly teleworking days may be linked to a higher likelihood of short SA, indicating that workplaces should pay special attention to their employees’ wellbeing and health.

## Introduction

Since the outbreak of COVID-19 in March 2020, the working life of knowledge workers occupying offices has undergone a tremendous change (1, 2). Those experts who usually do office-based jobs with computers were first assigned to work remotely. Since 2022, while the waves of the pandemic started to subside, knowledge workers were allowed to return to offices. At the beginning of COVID-19, the rates of sickness absence (SA) decreased from pre-pandemic levels. That was due to closed healthcare services or limited access to healthcare services for conditions other than urgent, health-threatening conditions (3), working remotely even while sick (4, 5), and due to potentially non-diagnosed symptoms or diseases, as they may have been perceived to add burden to the overloaded healthcare system (6, 7). Although earlier studies of SA and telework among office workers exist, studies with an evaluation of the post-pandemic era are rare, as most have focused on the years before the pandemic (8) or the first years of the pandemic (9–11). Furthermore, they have had, e.g., a cross-sectional design, survey data, or both (10, 12–14), while studies assessing telework and/or SA with objective, employer-owned register data in longitudinal settings are rare (11). Thus, the knowledge of SA rates during and after COVID-19 among teleworking knowledge workers is lacking. Another aspect in earlier studies is that telework was measured at one time point only, or by surveying participants on whether they can work remotely or not (12). Although surveys are feasible for gathering large-scale data from thousands of individuals, they are hampered by reporting and memory biases. Furthermore, estimating telework by the survey is limited to a pre-specified timespan only (e.g., from the survey date to previous days, weeks, or months). Thus, more detailed and longitudinal measures of telework, together with SA evaluations, would be warranted, as various methods to track or collect daily location data are available (15, 16).

In general, SA can be considered as a proxy for health status and as an indicator of workability, since short (1–3 or 1–5 days by self-certification) SA (later referred to as shortSA) is known to predict longer SA (17, 18) (later referred to as longSA). The shortSA by self-certification is common in Nordic countries, and they are compensated by employers in Finland. These shortSA are mainly used for health-related reasons, such as epidemics or for symptoms such as coughing, headache, or migraine that prevent employees from working or might be contagious, for example, the stomach flu. In addition, shortSA may represent different causes than longSA, which requires medical certification by a physician. Moreover, longSA is compensated by the social insurance in Finland and other Nordic countries. Some evidence exists that shortSA reflects self-perceived health with or without underlying disease (19). Thus, investigating shortSA and telework is needed to understand the linkage between working conditions (remote work vs. working at the employer’s premises in this study) and SA.

This prospective cohort study aimed to investigate (1–3 days) shortSA before, during, and in the follow-up to the COVID-19 pandemic among a cohort of Finnish knowledge workers. Another aim was to investigate the association of telework days/week for shortSA. We hypothesized that the increasing number of telework days would be associated with a lower likelihood of shortSA.

## Method

**Figure 1:**
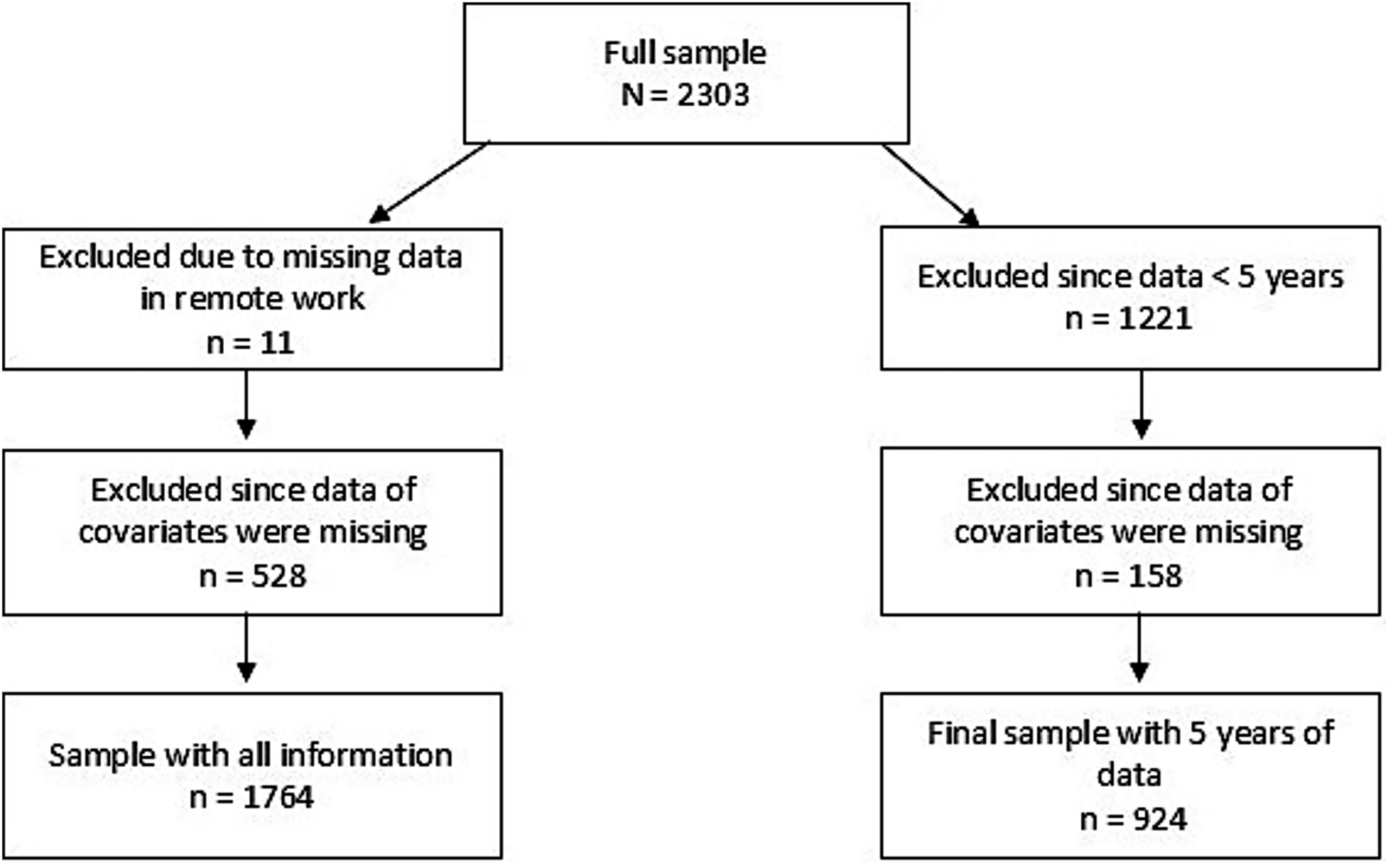
Flow chart of the sample definition.

For this register-based prospective study, we first included all employees with employer-owned register data for SA and telework (*N* = 2,303, four organizations) for 2019–2023. The sizes of public and private organizations with mainly knowledge work varied from 250 to >1,000 employees. We defined the study sample by limiting the data to those without missing data. That resulted in a full sample of 1764 employees. Limiting the data further to those with 5 years of data resulted in a final sample of *n* = 924 employees (Figure 1).

The individual-level data were collected from the employers’ electronic records of working hours, work locations, and SA from the beginning of 2019 to the end of 2023, covering all employees employed in the participating organizations during that period. The working hours and locations data included the starting and ending times of daily working hours, working at the employer premises or remotely, travel due to work, and the reasons for any absences (day off, SA, maternity leave, annual leave, etc.), but also separate records of SA without any health-related information were collected (the start and end date for SA spells). Although the data were employer-owned, the working hours and locations data were initially entered into the tracking systems by the employees themselves. However, as the working hours’ data were used as a basis for salary in each participating organization, it can be assumed to be valid. For this study, potential duplicate entries for working hours and locations were removed, and any overlapping entries were evaluated, i.e., if there were two entries for a date, it was checked if they were partial duplicates or subsequent entries (<5% of all observations). For partial duplicates, i.e., with the same start or end time, or a difference in work location (premises vs. remote), the first entry was selected. That was done to avoid any interpretations based on the data and to treat observations systematically. In case of subsequent entries, i.e., reporting a new start time for work after an already entered end time, we utilized a limit of 1 h. Thus, if the time between two subsequent entries was ≤1 h, they were combined into one entry, and working hours were calculated for this complete period as has been done, e.g., in studies of working hours in shiftwork (20). This decision did not affect the estimates of telework, as we assigned a date for telework if full or partial working hours were entered as telework. Regarding the SA, we focused on the (1–3 days of self-certified) shortSA, calculating the number of shortSA days and all SA days (all lengths, later referred to as allSA). The SA data consisted of full-day absences confirmed by employers. To estimate seasonal variation and the effects of COVID-19 restrictions, we calculated the SA measures for each week from 2019 to 2023. We calculated the number of shortSA spells/week in each year for sensitivity analyses.

As a factor of interest, we calculated the number of days worked remotely per week based on the employer-owned register data.

The covariates accounted for in the analyses were available from the employer registers, including age (continuous), sex (woman vs. man), job title (working in an assistant role, being an expert, or as a team leader or supervisor), work experience in the organization, and working hours. Besides the employee-level covariates, we also included organization in the models to account for the potential effects of organizational culture and instructions for SA and telework. Although we obtained the covariates from the employer registers, they were not complete for the full sample or the final sample. To account for the missing covariate data, we chose not to impute but to add the covariates to the models step-by-step to test the effects on their associations. The covariates were selected based on the known associations with the outcome (shortSA) (21) and factor of interest (telework) (22), and they were thus controlled in the analyses.

This study was approved by the Ethical Board of the Finnish Institute of Occupational Health, Helsinki, Finland.

**Figure 2:**
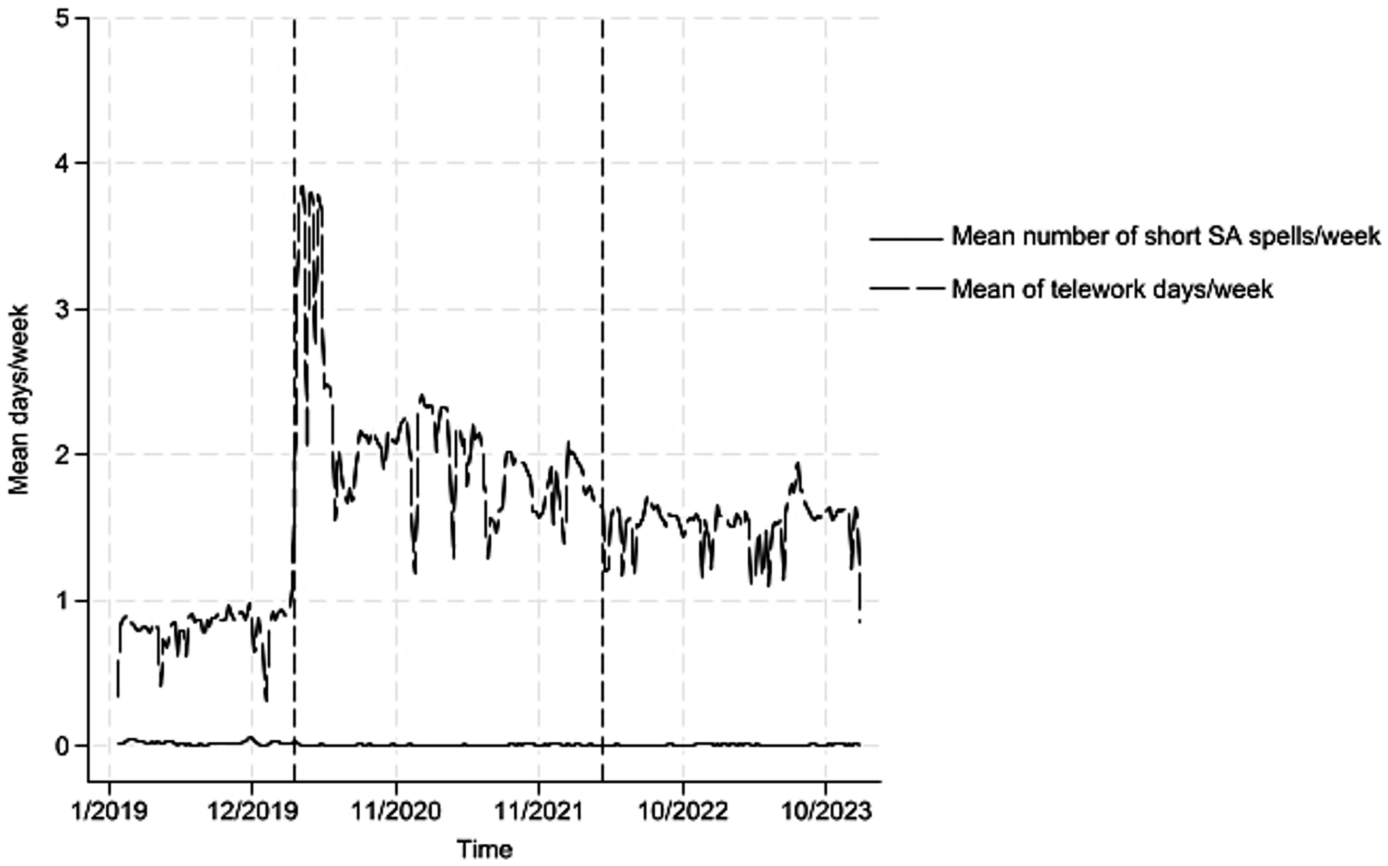
Means of short sickness absence days and telework days per week from 2019 to 2023 in the full sample of 1764 employees. The dashed vertical line indicates that COVID-19 restrictions started in Finland in March 2020 and ended in March 2022.

All statistical analyses were conducted with Stata 18 MP. Descriptive characteristics for the full sample (*n* = 1764) were calculated first for means with standard deviations (SD) or frequencies with percentages (%). Then we estimated the means across 2019–2023 for telework days/week and shortSA days/week (the maximum number of short SA days could be 5 days if two subsequent spells of shortSA took place during a week) and presented them graphically for the full sample of 1764 employees (Figure 2). Next, we utilized conditional Poisson regression for a longitudinal design with fixed-effects (fe option) to account for the repeated nature of observations in the data while using the final sample with 5 years of data (*n* = 924). The FE model was chosen as it accounts for within-individual changes in shortSA over the study period. The FE controls all stable characteristics of individuals, whether measured or not. The models included a crude model to adjust weekly working hours. Then, we added other influential factors to the models to obtain adjusted incidence rate ratios (IRRs) with 95% confidence intervals (CI). Supplementary Table S1 reports on the associations of covariates with shortSA. As a sensitivity analysis, we repeated the models using shortSA spells/week as an outcome.

## Results

The full study sample (*n* = 1764) with complete data had 74% women, were on average 48.9 years of age, had 67% temporary positions, and 62% had an expert-level job title (Table 1).

**Table 1:** Descriptive characteristics of the full sample (*n* = 1724).

	Mean	SD
Age (years)	48.9	10.1
Work experience (years)	15.9	11.0

	*n*	%
Sex		
Women	1,226	74
Men	426	26
Work contract		
Permanent	645	33
Temporary	1,305	67
Job title		
Assistants	194	19
Experts	646	62
Team leaders or supervisors	194	19

Figure 2 indicates the mean weekly levels of shortSA days and telework days for 2019 to 2023. The figure using shortSA spells showed the same pattern (data not shown).

The associations between teleworking and shortSA were tested step-wise to understand the effects of various covariates (Table 2). In the crude model, where we only accounted for weekly working hours and organization, each 1-day increase in telework was associated with a lower likelihood of shortSA in that week. However, accounting for age, sex, and work contract changed the direction and magnitude of the association toward a higher likelihood of shortSA that week. Adding the job title attenuated the association, while adding work experience had no further influence. Instead, adding allSA days as they would mean that an employee might have a health issue and potentially be away from work, the model, including all covariates, retained the association at the IRR 1.26 (95%CI 1.15, 1.38). The sensitivity analyses for shortSA spells/week retained the associations’ direction and magnitude (Supplementary Table S1).

**Table 2:** Conditional poisson regression models for associations between telework days/week and short sickness absence days/week with incidence rate ratios (IRR) with 95% confidence intervals (CI) among the final sample of 924 employees.

	Crude model		Adjusted model 1		Adjusted model 2		Adjusted model 3		Adjusted model 4	
	IRR*	95%CI	IRR*	95%CI	IRR*	95%CI	IRR*	95%CI	IRR*	95%CI
Telework days/week	0.91	0.89, 0.92	1.72	1.61, 1.84	1.32	1.21, 1.44	1.26	1.15, 1.38	1.35	1.22, 1.50

*Crude model adjusted with weekly working hours and organization; adjusted model 1 additionally adjusted for age, sex, and work contract; adjusted model 2 additionally adjusted for job title; adjusted model 3 additionally adjusted for all sickness absence days, and adjusted model 4 additionally adjusted for work experience.

## Discussion

This cohort study of 924 Finnish knowledge employees in public and private organizations aimed to investigate the rates of shortSA before, during, and in the follow-up to the COVID-19 pandemic, and the potential effects of telework on shortSA rates. To the best of our knowledge, such longitudinal studies with objective, employer-owned electronic data on telework and shortSA are rare.

The estimated rates of shortSA were low and showed no variation across follow-up from 2019 to 2023. Instead, the rates of telework increased dramatically in March 2020 when COVID-19 restrictions were first applied. Although teleworking has remained higher than before the COVID-19 pandemic, most knowledge workers also work at employer’s premises. Another aim was to estimate the associations between telework and shortSA, indicating that employee characteristics, including age, sex, work contract, job title, and work experience, played an important role in the associations. Each 1-day increase in telework days/week seemed to increase the likelihood of shortSA in the model accounting for the covariates, while in the crude model, the association was the opposite, i.e., toward lower likelihood. This finding was robust for the shortSA measure as the associations retained magnitude and direction when using shortSA spells/week as an outcome. All in all, our results might also imply that as shortSA is based on self-assessed sickness, i.e., calling in as sick, it might also reflect the fact that telework may enable working even while sick (23, 24). Thus, further studies should assess this masking effect.

### COVID-19 restrictions have affected telework

All in all, the COVID-19 pandemic was an exceptional situation in working life as national regulations were applied in response to acute decisions to safeguard the population. In Finland, a lockdown was applied in March 2020, and social distancing, including a directive for telework, was imposed. These measures raised concerns about employee perceptions and strain (1, 2, 25), while such directives were in action until April 2022. As telework has shown positive and negative associations with employee wellbeing and health (26, 27), and a higher number of weekly teleworking days has been related mainly to positive outcomes (28). However, the linkage with shortSA has remained important to be shown since the earlier studies of allSA and telework among office workers have focused on the years before the pandemic (8) or the first years of the pandemic (9–11).

### Comparison of the current study with earlier research

Our results add to the existing knowledge of the associations between telework and shortSA that have been rarely investigated and mainly based on the early COVID-19 pandemic (9–11), or years before the pandemic (8). Furthermore, our results based on the mean levels of shortSA and telework across 2019–2023 add to the existing literature, indicating that no such peaks in shortSA could be seen. Furthermore, the associations between telework and SA indicate that both employee (e.g., age and sex) and work-related factors (e.g., job title and work contract) play an influential role. That aligns with earlier findings for telework (26, 27, 29) and allSA (21, 30), suggesting that such factors may affect the associations between telework and SA, as shown in our crude and adjusted models. Although the social distancing and restriction directives have taken place globally, we are aware that compensated allSA or shortSA is not. Thus, our results might be most generalizable to Finland but potentially apply to the Nordic countries with similar welfare models and working life as to other countries. The same may apply to telework, as some indications exist that the transition to telework was exceptional in Finland, with high coverage of high-level IT technology (25).

### Strengths and limitations

The strength of this study was the use of employer-owned register data of employees, including working hours, teleworking, shortSA, and other characteristics. Such data of all the employees in the organization are objective, accurate, and free from biases relating to memory, self-reporting, or obtaining consent. Further strengths were a rather large sample, 1764 employees with complete data on teleworking, shortSA, and covariates, and even the final sample of 924 employees with 5 years of follow-up. Furthermore, our sample of knowledge employees in the public and private sectors is comparable to the general population in Finland working in similar occupations (25). Despite the benefits of the register data, we still had some missing data. That is due to the turnover of employees due to a change of workplace, retirement, or even other longer absences (due to, e.g., parental leaves, work incapacity, or else). Since we estimated the mean levels of telework and SA both for the full sample (Supplementary Figure S1) and for those with 5 years of data (Figure 1), we could not detect any visible differences or trends. However, further studies could address the turnover by using partitioned follow-up or other statistical methods accounting for censoring. Many measures of SA exist, and we applied shortSA in this study as it was expected to reflect the epidemics and/or work burden since longer absences require a certificate from a medical expert. We used two measures, shortSA days and spells, to test the findings. The results were also tested for allSA days (i.e., regardless of the length), indicating that the results hold. Thus, our results should be robust for the availability of healthcare during the pandemic. Of course, one can argue that the time after COVID-19 is not yet available, but we consider the possibility of investigating 5 years of teleworking and shortSA rather unique, especially using register data. Furthermore, our use of employer-owned register data might be a drawback due to limited access to relevant covariates. Based on the earlier research, mental health, work-life balance, or perceived work environment may play a role in the associations between telework and SA (31, 32), why should further studies pay attention to them.

To conclude, the peak of telework during the lockdown due to the COVID-19 pandemic in March 2020 has remained at an elevated level among Finnish knowledge workers until the end of 2023. No peaks or variations in levels of shortSA were detected. Working more days remotely was associated with a higher likelihood of shortSA while accounting for employee and work-related factors. The maintenance of wellbeing and health in telework might need special attention from supervisors and guidelines from workplaces.

## Data Availability

The datasets presented in this article are not readily available because data will be shared on reasonable request to the corresponding author with permission of the participating organizations. Requests to access the datasets should be directed to annu.haapakangas@ttl.fi.

## References

[1] van Zoonen W Sivunen A Blomqvist K Olsson T Ropponen A Henttonen K . Factors influencing adjustment to remote work: employees' initial responses to the COVID-19 pandemic. Int J Environ Res Public Health. (2021) 18:6966. doi: 10.3390/ijerph18136966, PMID: 34209796 PMC8297254

[2] van Zoonen W Sivunen A Blomqvist K Olsson T Ropponen A Henttonen K . Understanding stressor–strain relationships during the COVID-19 pandemic: the role of social support, adjustment to remote work, and work–life conflict. J Manag Organ. (2021) 27:1038–59. doi: 10.1017/jmo.2021.50

[3] Van Der Feltz-Cornelis CM Varley D Allgar VL de Beurs E. Workplace stress, Presenteeism, absenteeism, and resilience amongst university staff and students in the COVID-19 lockdown. Front Psych. (2020) 11:588803. doi: 10.3389/fpsyt.2020.588803, PMID: 33329135 PMC7728738

[4] Ruhle SA Schmoll R. COVID-19, telecommuting, and (virtual) sickness Presenteeism: working from home while ill during a pandemic. Front Psychol. (2021) 12:734106. doi: 10.3389/fpsyg.2021.734106, PMID: 34721202 PMC8554096

[5] Ferreira AI Mach M Martinez LF Miraglia M. Sickness Presenteeism in the aftermath of COVID-19: is Presenteeism remote-work behavior the new (ab)normal? Front Psychol. (2022) 12:748053. doi: 10.3389/fpsyg.2021.748053, PMID: 35153891 PMC8830031

[6] Lyttelton T Zang E. Occupations and sickness-related absences during the COVID-19 pandemic. J Health Soc Behav. (2022) 63:19–36. doi: 10.1177/00221465211053615, PMID: 35100514 PMC9013443

[7] Smith DRM Jijón S Oodally A Shirreff G Aït Bouziad K Ante-Testard PA . Sick leave due to COVID-19 during the first pandemic wave in France, 2020. Occup Environ Med. (2023) 80:268–72. doi: 10.1136/oemed-2022-108451, PMID: 36914254 PMC10176331

[8] Nielsen MB Knardahl S. The impact of office design on medically certified sickness absence. Scand J Work Environ Health. (2020) 46:330–4. doi: 10.5271/sjweh.3859, PMID: 31647108

[9] Biron C Karanika-Murray M Ivers H Salvoni S Fernet C. Teleworking while sick: a three-wave study of psychosocial safety climate, psychological demands, and Presenteeism. Front Psychol. (2021) 12:734245. doi: 10.3389/fpsyg.2021.734245, PMID: 34777119 PMC8581213

[10] Grzelczak A. Remote work and its consequences for the employee in the time of the Covid-19 pandemic. Eur Res Stud J. (2021) 24:399–411. doi: 10.35808/ersj/2740, PMID: 40697737

[11] Lancaster K Tumin D Campbell KM. Changes in sick leave utilization at an academic medical center during the COVID-19 pandemic. Int J Workplace Health Manag. (2023) 16:145–56. doi: 10.1108/IJWHM-02-2022-0025

[12] Borge RH Johannessen HA Fostervold KI Nielsen MB. Office design, telework from home, and self-certified sickness absence: a cross-sectional study of main and moderating effects in a nationally representative sample. Scand J Work Environ Health. (2023) 49:222–30. doi: 10.5271/sjweh.4078, PMID: 36645881 PMC10621899

[13] Aloisi A De Stefano V. Essential jobs, remote work and digital surveillance: addressing the COVID-19 pandemic panopticon. Int Labour Rev. (2022) 161:289–314. doi: 10.1111/ilr.12219, PMID: 34548685 PMC8444901

[14] Gerich J. Home-based telework and Presenteeism: new lessons learned from the Covid-19 pandemic. J Occup Environ Med. (2022) 64:243–9. doi: 10.1097/JOM.0000000000002414, PMID: 34654037 PMC8887682

[15] Olde Kalter M-J Geurs KT Wismans L. Post COVID-19 teleworking and car use intentions. Evidence from large scale GPS-tracking and survey data in the Netherlands. Transp Res Interdiscip Perspect. (2021) 12:100498. doi: 10.1016/j.trip.2021.100498, PMID: 34909635 PMC8661099

[16] Ropponen A Harma M Bergbom B Natti J Sallinen M. The vicious circle of working hours, sleep, and recovery in expert work. Int J Environ Res Public Health. (2018) 15:1361. doi: 10.3390/ijerph15071361, PMID: 29958458 PMC6068518

[17] Hultin H Lindholm C Malfert M Moller J. Short-term sick leave and future risk of sickness absence and unemployment - the impact of health status. BMC Public Health. (2012) 12:861. doi: 10.1186/1471-2458-12-861, PMID: 23050983 PMC3508966

[18] Laaksonen M He L Pitkaniemi J. The durations of past sickness absences predict future absence episodes. J Occup Environ Med. (2013) 55:87–92. doi: 10.1097/JOM.0b013e318270d724, PMID: 23235465

[19] Marmot M Feeney A Shipley M North F Syme SL. Sickness absence as a measure of health status and functioning: from the UK Whitehall II study. J Epidemiol Community Health. (1995) 49:124–30. doi: 10.1136/jech.49.2.124, PMID: 7798038 PMC1060095

[20] Harma M Ropponen A Hakola T Koskinen A Vanttola P Puttonen S . Developing register-based measures for assessment of working time patterns for epidemiologic studies. Scand J Work Environ Health. (2015) 41:25788103:268–79. doi: 10.5271/sjweh.3492 25788103

[21] Allebeck P Mastekaasa A. Swedish council on technology assessment in health care (SBU). Chapter 5. Risk factors for sick leave - general studies. Scand J Public Health Suppl. (2004) 63:49–108. doi: 10.1080/14034950410021853 15513654

[22] Charalampous M Grant CA Tramontano C Michailidis E. Systematically reviewing remote e-workers’ well-being at work: a multidimensional approach. Eur J Work Organ Psychol. (2019) 28:51–73. doi: 10.1080/1359432X.2018.1541886

[23] Steidelmüller C Meyer S-C Müller G. Home-based telework and Presenteeism across Europe. J Occup Environ Med. (2020) 62:998–1005. doi: 10.1097/JOM.0000000000001992, PMID: 32796258 PMC7720871

[24] Patel C Biron M Cooper SC Budhwar PS. Sick and working: current challenges and emerging directions for future presenteeism research. J Organ Behav. (2023) 44:839–52. doi: 10.1002/job.2727

[25] Blomqvist K Sivunen A Vartiainen M Olsson T Ropponen A Henttonen K . Remote work in Finland during the Covid-19 pandemic. Results of a longitudinal study. Lappeenranta: Lappeenranta-Lahti University of Technology (2020)

[26] Fan W Moen P. Ongoing remote work, returning to working at work, or in between during COVID-19: what promotes subjective well-being? J Health Soc Behav. (2023) 64:152–71. doi: 10.1177/00221465221150283, PMID: 36694978 PMC9902780

[27] Lyzwinski L-N. Organizational and occupational health issues with working remotely during the pandemic: a scoping review of remote work and health. J Occup Health. (2024) 66:uiae005. doi: 10.1093/joccuh/uiae005, PMID: 38289710 PMC11069417

[28] Gajendran RS Ponnapalli AR Wang C Javalagi AA. A dual pathway model of remote work intensity: a meta-analysis of its simultaneous positive and negative effects. Pers Psychol. (2024) 77:1351–86. doi: 10.1111/peps.12641

[29] Prasad K Mangipudi MR Vaidya R Muralidhar B. Organizational climate, opportunities, challenges and psychological wellbeing of the remote working employees during COVID-19 pandemic: a general linear model approach with reference to information technology industry in Hyderabad. Int J Adv Res Eng Technol. (2020) 11:372–89.

[30] Piirainen H Räsänen K Kivimäki M. Organizational climate, perceived work-related symptoms and sickness absence: a population-based survey. J Occup Environ Med. (2003) 45:175–84. doi: 10.1097/01.jom.0000052957.59271.f4, PMID: 12625232

[31] Haapakangas A Ropponen A Tulenheimo-Eklund E Ruohomäki V Reijula K. Associations of perceived privacy at the workplace with short sickness absences in a cohort of Finnish office workers. J Occup Environ Med. (2025) 67:e127–31. doi: 10.1097/JOM.0000000000003287, PMID: 39667748 PMC11801456

[32] Asmussen KE Mondal A Bhat CR Pendyala RM. On modeling future workplace location decisions: an analysis of Texas employees. Transp Res A Policy Pract. (2023) 172:103671. doi: 10.1016/j.tra.2023.103671, PMID: 41181831

