# Supplementary material for: The impact of the COVID-19 pandemic on telework and short sickness absences among Finnish knowledge workers
Source: Front Public Health. 2025 Nov 19;13:1683731. doi: 10.3389/fpubh.2025.1683731 (PMC12672299; doi:10.3389/fpubh.2025.1683731)
Supplement: Supplementary file 1 [file Table_1.docx]

**Supplemental Table S1** Conditional Poisson regression models for associations between telework days/week, covariates, and shortSA days/week with incidence rate ratios (IRR) with 95% confidence intervals (CI) among the final sample of 924 employees

|  | **IRR** | **95%CI** |
| --- | --- | --- |
| Telework days/week | 1.35 | 1.22, 1.50 |
| Age (years) | 0.96 | 0.93, 0.98 |
| Work experience (years) | 0.99 | 0.97, 1.01 |
| Sex (reference being women) |  |  |
| Men | 0.80 | 0.51, 1.25 |
| Work contract (reference permanent) |  |  |
| Temporary | 13.69 | 4.92, 38.06 |
| Job title (reference experts) |  |  |
| assistants | 1.02 | 0.98, 1.06 |
| team leaders or supervisors | 0.98 | 0.95, 1.03 |
