# Supplementary figures and images for: The impact of the COVID-19 pandemic on telework and short sickness absences among Finnish knowledge workers
Source: Front Public Health. 2025 Nov 19;13:1683731. doi: 10.3389/fpubh.2025.1683731 (PMC12672299; doi:10.3389/fpubh.2025.1683731)

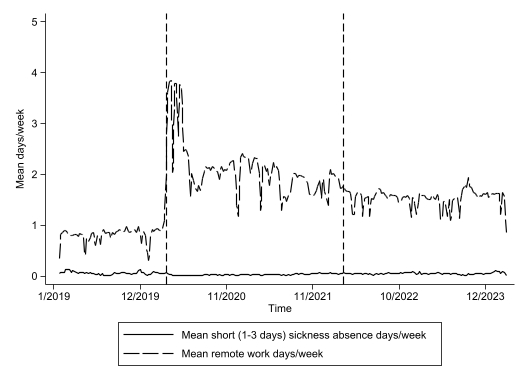

Supplement: Supplementary file 2 [file Image_1.jpeg]
